# Two cases of successful pregnancy in patients with Gitelman’s syndrome 

**DOI:** 10.5414/CN108526

**Published:** 2015-08-07

**Authors:** Dia R. Waguespack, Riyaj Kasekar, Khaled Abdel-Kader, Rachel B. Fissell

**Affiliations:** 1 Division of Nephrology, Vanderbilt University Medical Center, Nashville, TN, and; 2 Division of Nephrology, University of Pittsburgh Medical Center, Pittsburgh, PA, USA

**Keywords:** Gitelman’s syndrome, pregnancy

## Abstract

Gitelman’s syndrome (GS) is a distal convoluted tubule (DCT) defect clinically characterized by hypokalemic metabolic alkalosis. Pregnancy in women with GS often results in severe hypomagnesemia and hypokalemia. We report two cases of successful pregnancies, after previous fetal loss, in patients with GS managed with aggressive oral and intravenous electrolyte repletion. These cases illustrate increased potassium and magnesium requirements over the course of the pregnancies and are notable due to the high doses of electrolytes required. They also demonstrate the possibility of successful pregnancy outcomes with frequent laboratory monitoring and aggressive titration of electrolyte replacement either orally or intravenously to maintain appropriate serum levels necessary to provide a suitable environment for fetal development.

## Introduction 

Gitelman’s syndrome (GS) is generally a distal convoluted tubule defect (DCT) caused by an inactivating mutation in the SLC12A3 gene that encodes the thiazide sensitive sodium chloride co-transporter (NCCT). The loss of function at this transporter is responsible for the observed metabolic derangements. Over 180 different putative loss-of-function mutations have been identified [1]. The gene is inherited in an autosomal recessive pattern with phenotypic variation. The disorder affects ~ 25 people per million [2]. Mutations in the SCL12A3 gene result in a clinical syndrome of hypokalemia, hypomagnesemia, hypocalciuria, and metabolic alkalosis [3]. 

Clinical effects are observed due to physiologic disturbances in the setting of the DCT malfunction. Without proper function of the NCCT there is increased delivery of sodium to the collecting duct and resulting mild volume contraction. This leads to activation of the renin angiotensin aldosterone system. The increased aldosterone in turn increases sodium reabsorption via the epithelial sodium channel (ENaC) with subsequent secretion of potassium and hydrogen ions [2]. Volume contraction is responsible for passive reabsorption of calcium and resultant hypocalciuria. Decreased epithelial magnesium channels, TRPM6, lead to the observed hypomagnesemia [4]. Together these changes can significantly alter the electrolyte homeostasis in an individual. 

The phenotypic penetrance and expressivity varies and thus the clinical presentation of affected patients varies. Symptoms can include muscle weakness, tetany, salt craving, thirst, nocturia, paresthesias, and abdominal pain [5]. In severe cases with greater phenotypic penetrance, electrolyte replacement, and resultant symptom management can be challenging for a clinician. These challenges are often amplified in the setting of pregnancy. Renal physiologic changes in pregnancy not complicated by GS include volume expansion, increased renal blood flow, an increase in glomerular filtration rate, and altered tubular function. These changes are due in part to altered hormonal balance from that of the pre-pregnancy steady state [6]. Many women with GS are unaware they have the disorder until it is unmasked by the physiologic changes in renal function caused by pregnancy. Pregnancy in a patient with GS may result in a dramatically increased need for electrolyte replacement to maintain safe levels. As GS is a rare disease, incidence of pregnancy in GS is not well defined. To date there are 25 reported cases in the literature [7, 8, 9, 10, 11, 12, 13, 14, 15, 16, 17, 18]. When pregnancy in a GS patient occurs, appropriate management of electrolytes can affect the outcome. We present two distinct patients with GS complicating their pregnancies. Both patients required aggressive electrolyte repletion and we propose suggestions for clinical management of similar challenging cases. 

## Case 1 

A 25-year-old woman with GS presented at 6 weeks gestation with asymptomatic hypokalemia and hypomagnesemia. This was her fifth pregnancy. The prior pregnancies had been unsuccessful with the first three resulting in spontaneous abortion before 20 weeks and the fourth pregnancy complicated by fetal demise at 23 weeks. Her initial diagnosis of GS was made after the first pregnancy based on clinical presentation and laboratory data. Pre-pregnancy, she was maintained on oral electrolyte replacement. 

Late into her first trimester she became symptomatic with three separate emergency room visits for abdominal cramping. Symptoms of nausea, vomiting, perioral paresthesias, and tetany were absent. Electrocardiogram was normal. At 10 weeks gestation, on an oral regimen of 60 mEq of potassium chloride and 2,400 mg (119 mEq) of magnesium oxide per day, she was unable to maintain serum potassium levels greater than 3 mEq/L and serum magnesium levels greater than 1 mg/dL. Due to her clinical symptoms, laboratory data, and prior failed pregnancies in the setting of electrolyte derangements, she was started on thrice-weekly intravenous (IV) potassium and magnesium supplementation, as an outpatient at a local hospital, in addition to her oral regimen. Nephrology was then consulted to further guide management of her care. 

**Figure 1.**
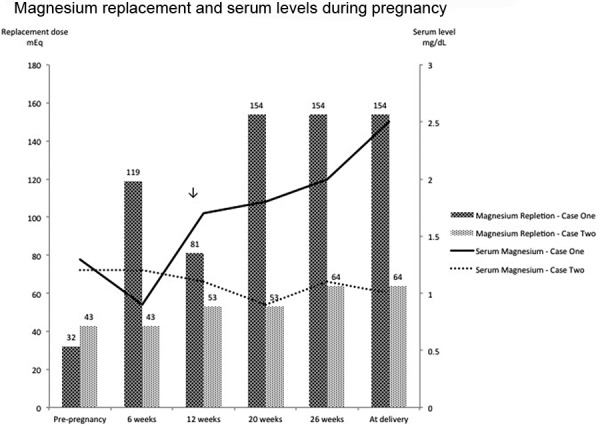
Magnesium supplementation (depicted by the bar graph) and corresponding week during pregnancy (dose in mEq is given above each bar). Serum magnesium levels (depicted by the line graph) and corresponding week during pregnancy. Downward arrow indicates patient 1’s transition from oral to intravenous supplementation.

**Figure 2:**
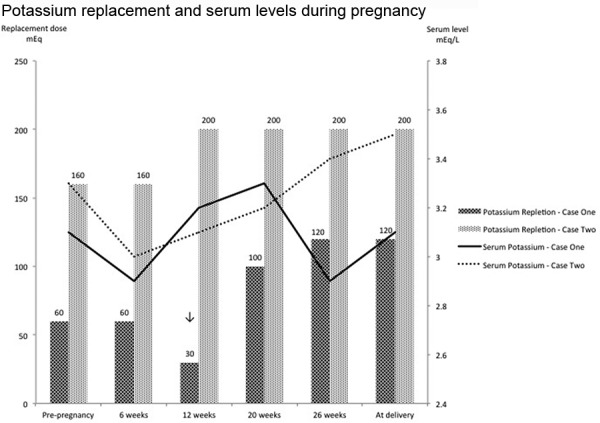
Potassium supplementation (depicted by the bar graph) and corresponding week during pregnancy (dose in mEq is given above each bar). Serum potassium levels (depicted by the line graph) and corresponding week during pregnancy. Downward arrow indicates patient 1’s transition from oral to intravenous supplementation.

Despite her aggressive oral regimen and thrice weekly IV supplementation she was unable to maintain a serum potassium greater than 3 mEq/L and a serum magnesium greater than 1.5 mg/dL. Outpatient, continuous IV electrolyte supplementation was started at 13 weeks gestation. Home health care services were arranged. She was started on a 24-hour infusion of magnesium and potassium. Initial dose of magnesium was 10 g of magnesium sulfate and 20 mEq potassium chloride. The electrolyte solution was reconstituted in 240 mL of normal saline and infused at a rate of 10 mL/h via continuous pump through a peripherally inserted central catheter. The patient had labs monitored initially twice a week and then once a week, with continuously increased doses of electrolyte solutions (Figure 1, Figure 2). With this regimen she was able to maintain serum potassium of 2.5 – 3.3 mEq/L, and serum magnesium of 1.7 – 2.5 mg/dL (normal lab range 1.6 – 2.6 mg/dL), close to our previously defined targets. Her maximum repletion requirements were 120 mEq of IV potassium and 19 g (154 mEq) of IV magnesium sulfate per day. At 28 weeks gestation, due to severe intrauterine growth restriction and reversed end-diastolic placental flow, she was hospitalized and delivered via cesarean section a viable, 636 g infant. 

## Case 2 

The second patient is an otherwise healthy 34-year-old woman referred to nephrology for evaluation of hypokalemia and hypomagnesemia. She was diagnosed with GS after presenting with a serum potassium of 2.6 mEq/L, serum magnesium of 0.9 mg/dL, metabolic alkalosis, modestly low blood pressure, and additional testing that revealed an elevated urine potassium and chloride, increased fractional excretion of magnesium, hypocalciuria, and negative diuretic screen. Interestingly, she had been maintained on an oral contraceptive pill containing drospirenone until shortly prior to her initial presentation for hypokalemia. Genetic testing was not pursued. 

One year later, at the time of her first pregnancy, she presented with serum potassium and magnesium levels of 2.9 mEq/L and 1.4 mg/dL, respectively. Her oral repletion was increased to 200 mEq/day of potassium chloride and 640 mg/day (53 mEq) of magnesium chloride. Spontaneous fetal demise occurred at 7 weeks and 6 days. Her serum potassium and magnesium levels were 3.6 mEq/L and 1.0 mg/dL at the time of this event. 

She became pregnant again 1 year later. At the time of her second pregnancy, oral potassium and magnesium replacement were increased from pre-pregnancy doses of potassium chloride 160 mEq/day and magnesium chloride 512 mg/day (43 mEq) to potassium chloride 200 mEq/day and magnesium chloride 768 mg/day (64 mEq). Serum potassium and magnesium levels were monitored at 3-week intervals throughout the pregnancy. Goal levels were achieved with a serum potassium of ~ 3.2 meq/L and serum magnesium levels greater than 1.0 – 1.1 mg/dL (normal lab range 1.5 – 2.5 mg/dL). She was asymptomatic throughout her pregnancy and her electrocardiogram was unremarkable. She delivered a healthy infant at 33 weeks gestation via cesarean section. 

## Discussion 

Although the exact cause for the substantial increased electrolyte need during pregnancy in patients with GS is not entirely clear, possible contributors can be inferred from the renal physiology of pregnancy and studies examining the effects of sex hormones on NCCT expression. Pregnancy results in an increase in GFR and renal plasma flow (RPF) along with an upregulation of the renin-angiotensin-aldosterone system [19]. In typical pregnancy, an increase in aldosterone would be postulated to produce a kaliuretic effect. However, this effect is not normally seen due to antagonism from progesterone [20]. Perhaps to mitigate the sodium loss from these antagonistic effects, estrogens are believed to upregulate NCCT expression [17]. In the presence of GS, such upregulation along with fetal demands and losses associated with hyperfiltration, may in part explain the drastic increased need for electrolyte replacement in our two cases and those reported in the literature [15, 21]. Although the exact mechanism remains unclear, tubular defects in GS clearly disrupt the normal physiologic adjustments to electrolyte handling, a stark contrast to unaffected pregnant women. 

Magnesium levels have been shown to decrease in normal pregnancy [22]. Although the precise physiologic reasons are not clear, increased magnesium demands are seen in patients with GS who then become pregnant. This is consistent with what was observed in our two cases and prior published literature. Murine models have shown fetal complications and increased mortality in pregnant mice that are deprived of dietary magnesium [23], suggesting that threshold magnesium levels are needed for successful fetal outcomes. 

Severe hypokalemia and hypomagnesemia also have potential adverse maternal health consequences. Electrolyte abnormalities may predispose to cardiac arrhythmias. Screening electrocardiograms should be obtained in all patients [2]. This becomes particularly important at time of delivery as pharmacologic therapy varies based on delivery method. Anesthetic complications can be avoided with careful consideration and monitoring of electrolytes and electrocardiography [10]. 

When presented with the clinical responsibility of the management of a pregnant patient with GS, crucial decisions related to electrolyte repletion must be made to ensure successful maternal and fetal outcomes. Past failed pregnancies may deter future reproductive planning. However, our cases and prior reports demonstrate that success is possible with aggressive management and close laboratory monitoring [24]. Reports present in the current literature suggest that close laboratory monitoring and aggressive electrolyte repletion can contribute to a successful pregnancy outcome [12]. The data at this time is all observational and anecdotal, and thus a causal association is suggested, but cannot be supported by the current literature. We acknowledge that the lack of laboratory information regarding the patient’s prior pregnancies prevents us from drawing a firm conclusion on whether aggressive electrolyte repletion and management alone explains her improved outcome in case 1. 

A choice of IV electrolyte replacement versus oral or some combination should be individualized based on the gestational course of the patient and the development of complications (e.g., hyperemesis gravidarum, symptomatic hypokalemia, etc.). Transition to IV electrolyte repletion should be considered when oral doses are not sustaining goal electrolyte levels or the side effect profile limits increased dosages. Based on our experience along with published cases, we recommend targeting a serum potassium level > 3 mEq/L and serum magnesium levels at or near the laboratory’s lower limit of normal [25]. Frequent laboratory monitoring is critical for appropriate dose adjustments. The growing fetus and the mother’s increasing progesterone and estrogen levels induce a dynamic state with changing electrolyte balance throughout the pregnancy. Based on our experience, we would suggest frequent lab monitoring, particularly in the beginning as initial goal levels are established. As pregnancy progresses, clinical judgment and patient response to therapy should guide interval laboratory monitoring. 

The use of the aldosterone antagonists, spironolactone and eplerenone, or the epithelial sodium channel inhibitor, amiloride, has been proposed. The aldosterone antagonists however are not widely used due to the theoretical risk of undervirilization of a male fetus. This risk may be lower with eplerenone due to its selectivity, but published experience is sparse. Spironolactone is pregnancy class D although there are reports in the literature of use during pregnancy without adverse effects [14, 26]. Eplerenone and amiloride are pregnancy class B, each with successful use reported in patients with GS [7, 8]. Due to the potential risks we chose not to use this therapy in our patients and demonstrated successful outcomes in both cases without potassium sparing diuretics. We recommend providers engage patients in a discussion that acknowledges the poorly characterized risk-benefit trade-offs of using these agents during pregnancy. 

GS is a rare disease, when present during pregnancy imparts additional management challenges and may be associated with an increased risk of maternal and fetal complications. The cases described are notable because of the high doses of electrolytes required. Both illustrate increased potassium and magnesium requirements over the course of the pregnancies, consistent with prior literature. They also demonstrate the possibility of successful pregnancy outcomes in patients with prior fetal loss. We recommend a treatment approach that involves frequent laboratory monitoring and aggressive titration of electrolyte replacement both orally and intravenously to maintain appropriate serum levels necessary to provide a suitable environment for fetal development and maternal health. Multidisciplinary management (e.g., maternal fetal medicine) of these challenging patients is critical to optimizing outcomes for mother and fetus. 

## Acknowledgment 

Dr. Abdel-Kader is supported by NIH grant K23DK090304. 

## Conflict of interest 

The authors have no conflicts of interest to disclose.
